# Mannose Binding Lectin, S100 B Protein, and Brain Injuries in Neonates With Perinatal Asphyxia

**DOI:** 10.3389/fped.2020.00527

**Published:** 2020-09-17

**Authors:** Cinzia Auriti, Giusi Prencipe, Rita Inglese, Maria Moriondo, Francesco Nieddu, Vito Mondì, Daniela Longo, Silvia Bucci, Tamara Del Pinto, Laura Timelli, Vincenzo Maria Di Ciommo

**Affiliations:** ^1^Neonatal Intensive Care Unit, Department of Medical and Surgical Neonatology, Bambino Gesù Children's Hospital, IRCCS, Rome, Italy; ^2^Laboratory of Rheumatology Department of Laboratories, Bambino Gesù Children's Hospital, IRCCS, Rome, Italy; ^3^Laboratory of Chemical Chemistry, Department of Laboratories, Bambino Gesù Children's Hospital, IRCCS, Rome, Italy; ^4^Laboratory of Immunology, Department of Pediatrics, Anna Meyer Children's University Hospital, Florence, Italy; ^5^Neonatology and Neonatal Intensive Care Unit, Policlinico Casilino Hospital, Rome, Italy; ^6^Neuroimaging Unit, Imaging Department, Bambino Gesù Children's Hospital, IRCCS, Rome, Italy; ^7^Clinical Psychology Unit, Department of Neurosciences, Bambino Gesù Children's Hospital, IRCCS, Rome, Italy; ^8^Unit of Epidemiology, Bambino Gesù Children's Hospital, IRCCS, Rome, Italy

**Keywords:** perinatal asphyxia, mannose binding lectin, genotype, S100B protein, therapeutic hypothermia

## Abstract

Perinatal asphyxia triggers an acute inflammatory response in the injured brain. Complement activation and neuroinflammation worsen brain damage after a systemic ischemia/reperfusion insult. The increase of mannose binding lectin (MBL) during asphyxia may contribute to the brain damage, via activation of the complement lectin pathway. The possible role of *MBL2* gene variants in influencing the severity of post-asphyxia brain injuries is still unexplored. This retrospective study included 53 asphyxiated neonates: 42 underwent therapeutic hypothermia (TH) and 11 did not because they were admitted to the NICU later than 6 h after the hypoxic insult. Blood samples from TH-treated and untreated patients were genotyped for *MBL2* gene variants, and biomarker plasma levels (MBL and S100 B protein) were measured at different time points: during hypothermia, during rewarming, and at 7–10 days of life. The timing of blood sampling, except for the T1 sample, was the same in untreated infants. Highest (peak) levels of MBL and *MBL2* genotypes were correlated to neuroimaging brain damage or death and long-term neurodevelopmental delay. *MBL2* wild-type genotype was associated with the highest MBL levels and worst brain damage on MRI (*p* = 0.046) at 7–10 days after hypoxia. MBL increased in both groups and S100B decreased, slightly more in treated than in untreated neonates. The progressive increase of MBL (*p* = 0.08) and to be untreated with TH (*p* = 0.08) increased the risk of brain damage or death at 7–10 days of life, without affecting neurodevelopmental outcomes at 1 year. The effect of TH on MBL plasma profiles is uncertain.

## Introduction

Perinatal asphyxia triggers an acute inflammatory response in the injured brain. Many studies (1–4) report an increase in circulating cytokine and chemokine levels in neonates with hypoxic ischemic encephalopathy, highlighting the role of neuroinflammation in brain damage. Reperfusion phenomena further increase the release of inflammatory substances, exacerbating injuries in multiple organs, regardless of the initial site (1–4). In neonates, the overexpression of inflammatory mediators could lead to both cerebral damage and poor prognosis (4–6). The effect of therapeutic hypothermia (TH) on the release of inflammatory mediators and their plasma levels remains poorly understood.

Mannose binding lectin (MBL) is a soluble receptor of the innate immune system. It increases in response to infections and can recognize and bind to specific terminal sugars (mannose and N-acetylglucosamine) located on the membrane of various pathogens. Upon binding, MBL has many important functions: it has opsonic and inflammatory actions, stimulates macrophage activation, facilitates phagocytosis, and promotes the lectin pathway of the complement system (7–9). The roles of innate immunity and MBL are crucial at birth, when adaptive immunity is not yet fully developed. Circulating MBL, like other acute phase proteins, increases in neonates during the first weeks of life (10), and low plasma MBL levels appear to increase the risk of infection in preterm and full-term neonates (11–14). MBL levels show a considerable genetic variability in humans, due to point mutations in both the promoter and exon domains of its gene, *MBL2*. The wild-type genotype promotes a robust inflammatory response and high plasma MBL levels after infectious and inflammatory stimuli. Mutations in both the promoter and exon-1 domains of *MBL2*, which affect ~20% of the Caucasian population, cause quantitative, and functional deficiency of circulating MBL in response to the same infectious and inflammatory stimuli. In healthy subjects, MBL appears to have other functions in addition to protecting against infections and debate continues as to the clinical relevance of low or high MBL levels in these subjects (15–18).

As observed with microorganisms, MBL recognizes and binds to cryptic self-antigens, which are exposed by cell death or ischemia–reperfusion tissue injuries. This binding activates the complement lectin pathway and exacerbates tissue damage (19, 20). MBL-deficient mice show smaller brain lesions after ischemia compared with wild-type mice (21, 22). In patients with MBL deficiency, stroke is characterized by smaller infarctions and less sequelae than in individuals with normal or high levels. This observation supports the hypothesis of a key role for high MBL levels in the pathophysiology of human stroke and in the severity of tissue damage secondary to ischemia–reperfusion insult (23–26).

Protein S100B is a calcium-binding protein, synthesized and secreted by astrocytes and Schwann cells. Although S100B is found mainly in glial cells, it is also present in many other types of cells outside the central nervous system. In healthy individuals, there are small amounts in cerebrospinal fluid, blood, and urine. Levels of S100B increase in many biological fluids (cerebrospinal fluid, blood, urine, and saliva) under pathological conditions (e.g., perinatal asphyxia, acute brain injury, neuroinflammatory/neurodegenerative disorders) and, in particular, when brain tissue is damaged. In such circumstances, high S100B levels are considered to be an indicator of cellular damage (27–30).

## Aims of the Study

We hypothesized that neonates carrying the wild-type genotype of the *MBL2* gene could have a worse neurological outcome after perinatal asphyxia as a result of high levels of circulating MBL and upregulation of the lectin complement pathway. Furthermore, TH, the current standard of care for mild neonatal asphyxia, could act by altering the plasma profile of inflammatory mediators. The aim of this retrospective study was therefore to explore the associations between the *MBL2* genotype, MBL phenotypes, and the severity of brain damage following neonatal asphyxia. We also evaluated the effect of TH on plasma levels of MBL and S100B protein in these patients.

## Methods

### Study Approvals

Parents provided written consent for admission of the newborn to the neonatal intensive care unit (NICU) and for use of patient's data for non-profit and anonymous scientific research. The Ethics Committee of the Bambino Gesù Children's Hospital (Protocol number: 1381 OPBG 2017- N°1381, 2017) approved the retrospective study.

### Patients

**Figure 1:**
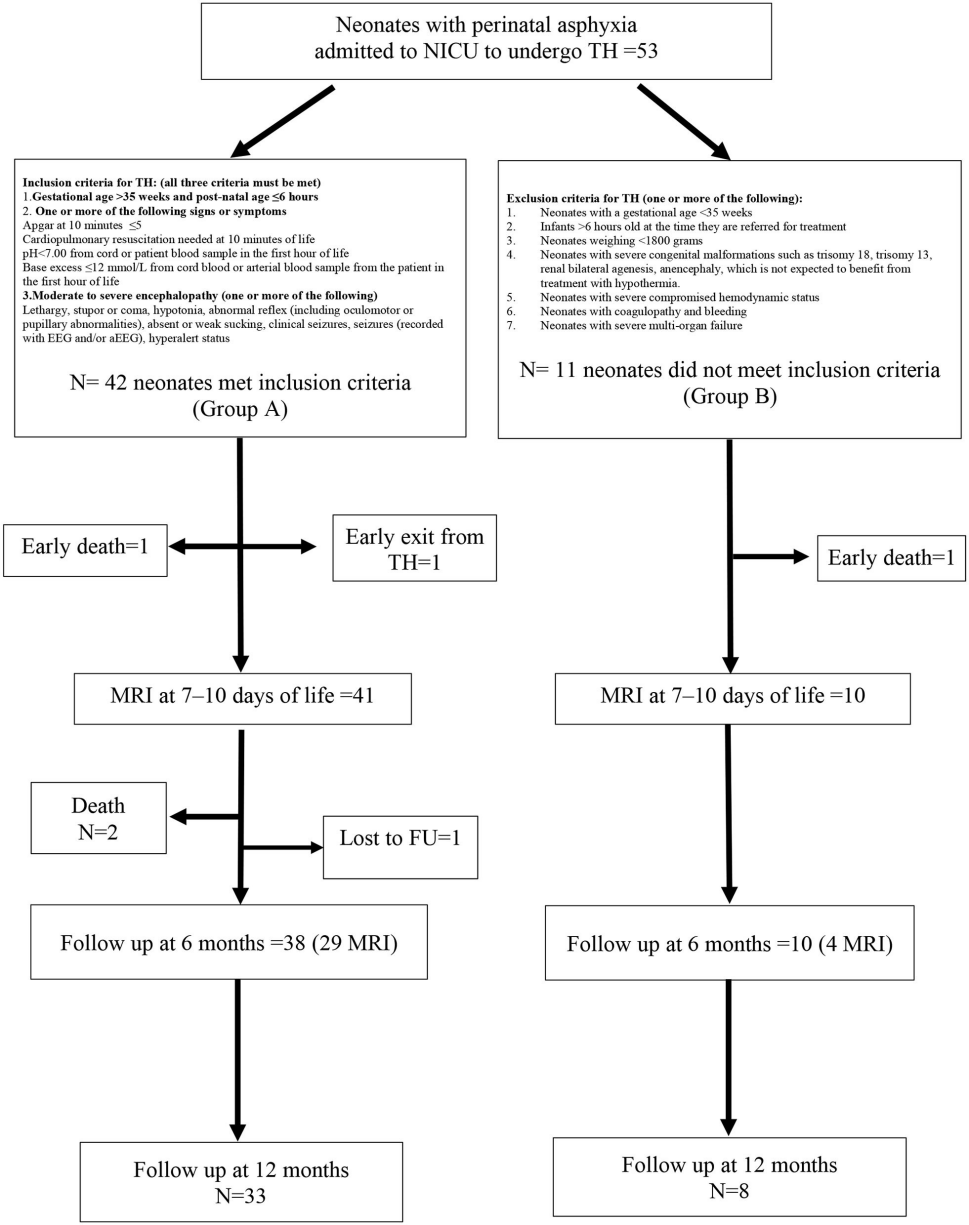
Study flowchart with inclusion and exclusion criteria for therapeutic hypothermia (TH). Forty-two neonates (group **A**) met inclusion criteria for hypothermia and 11 neonates (group **B**) were out of the temporal window and were untreated.

Out-born neonates (≥35 weeks and birth weight ≥1,800 g) could be included in the study if they had experienced moderate/severe perinatal asphyxia and were admitted to our NICU center to undergo total body TH. The study flowchart, definition of asphyxia, and inclusion/exclusion criteria for HT are shown in Figure 1. Mortality at any time from entry was not an exclusion criterion.

### Sample Collection and Analysis

As per routine clinical practice, blood samples had been collected from all neonates at the following time points after birth: 0–24 h (T1), 25–48 h (T2), 49–72 h (T3), and 7–10 days (T4), and then stored at −80°C until processing. Laboratory measurements and genotype analysis for the current study were carried out on residues of these routine blood samples. For the neonates treated with HT, the T1 values were based on samples taken during the first 6 h of life, before cooling was started.

### MBL and S100B Plasma Levels

MBL and S100B plasma levels were measured using ELISA (MBL oligomer, ELISA, Antibody Shop, Copenhagen, DK, and Biovendor), according to the manufacturer's instructions. In order to evaluate the possible association between high MBL levels and neurological damage, the analysis was based on peak levels of MBL and S100B, irrespective of when these occurred during the first 10 days of life and regardless of the precipitating event (i.e., ischemia or reperfusion).

#### MBL2 Genotyping

Genomic DNA was extracted from neonatal whole-blood samples using QIAmp DNA Blood kit (Quiagen, Germany), according to the manufacturer's instructions. Genotypes of MBL2 exon-1 (codons 52, 54, and 57) and Y/X promoter (−290G>C) polymorphisms were identified by PCR-RFLP analysis. The exon-1 wild-type allele was designated as A, while mutants in codons 54, 57, and 52 were collectively referred to as O and designated separately as B, C, and D, respectively. The wild-type promoter allele was designated as Y and the mutant was designated as X.

Genotyping analyses were performed in the immunology laboratory at Anna Meyer Children's Hospital, which is part of the Leonardo's Program Quality VEQ (external quality control), (https://www.abanalitica.it/?link=dettaglio&ID=3). All genetic screenings were performed in duplicate, and rare genotypes were subject to further analysis. In all analyses, samples with known genotypes were included as positive controls.

### Clinical Management During Hospitalization and After Discharge

#### Management During Hypothermia

During TH, neonates were cooled to maintain their core temperature at 33.5°C (accepted range, 33–34°C) for 72 h, followed by rewarming by 0.5°C/h over 6 h. During hypothermia, neonates underwent routine continuous amplitude-integrated electroencephalography (aEEG; Olympic CFM™6000 aEEG Infant Monitor) and/or video electroencephalography monitoring. Medical management included maintenance of euglycemia, fluid intake restriction, ventilatory support when necessary, vasopressor support, and timely treatment of coagulopathy and seizures.

### Neuroimaging

All enrolled neonates, who survived until the 7–10th day of life, had a brain magnetic resonance imaging (MRI) within this time frame (51/53 patients). The magnetic resonance protocol (3-T Skyra, Siemens, Erlagen, Germany) included axial T1 SE, sagittal T1 TIRM, axial and coronal T2 TSE, and DWI with a single-shot echo-planar sequence. Additional sequences were axial T2 GRE and axial T2 FLAIR. Pathological lesions were classified by a Pediatric Neuroradiologist according to the Basal Ganglia/Watershed (BG/WS) Barkovich score (31) (Supplemental File 1).

### Management After Discharge From NICU

Neonates who survived were monitored during a 1-year follow-up period (Supplemental File 2). Each child had a multidisciplinary assessment, consisting of serial clinical neurological examinations (32), vision and hearing evaluation, child development assessment using Bayley Scales of Infant Development III (33), neuroimaging, brain ultrasound exams, and MRI at 6 and 12 months of life, when appropriate.

Adverse neurological outcomes were defined as the presence of pathological impairment on brain MRI (BG/WS Barkovich scores of 1–4), severe neurological impairment, use of anticonvulsant therapy, and/or a Mental Development Index score ≤ 70 at 6–12 months.

### Statistical Analysis

For continuous variables, mean and standard deviation or median and interquartile range were presented. For categorical variables, absolute, and relative frequencies were presented. Characteristics at admission to NICU (baseline) were stratified by TH (yes/no) and compared using non–parametric tests (Fisher's exact test for categorical variables and Wilcoxon test for continuous variables). Mean values and 95% confidence intervals (CIs) for MBL and S100B levels at different time points were estimated using mixed linear regression models. In certain models, time points and hypothermia treatment group were included as fixed covariates. Data were clustered by patient and a random intercept was used. For S100B and for outcome measures showing greatest variability, log-transformed data were analyzed, with results that were similar to those obtained with the raw data.

To evaluate variables associated with the severity of brain damage observed on MRI, ordered logistic regression was applied with the following variables as covariates: treatment group, seizures before and during hospitalization, pH value, base excess, and peak levels of MBL and S100B during the first 10 days of life. Relationships between *MBL2* genotypes and short-term and long-term outcomes were also explored. Finally, to evaluate the association between hypothermia and neurological outcomes at 6–12 months, the incidence rate ratio (IRR) with 95% CI was estimated; *p* < 0.05 was considered statistically significant. All of the analyses were performed using Stata version 13.

## Results

### General Data

In total, 53 neonates were enrolled in the study, 42 of whom received HT (Group A). The remaining 11 patients (Group B) did not receive HT as they were admitted >6 h after the hypoxic insult. The characteristics of the study population, stratified by hypothermia, are summarized in Table 1. The two groups differed in age at admission, intubation before admission to the NICU, and Sarnat score at birth, with all of the data indicating that neonates treated with HT presented with a more serious clinical condition compared with the untreated neonates. Two neonates (one in Group A and one in Group B) deceased before an MRI could be performed at 7–10 days of life. One neonate (Group A) developed an intractable coagulopathy, which resulted in premature discontinuation of hypothermia and then he deceased (Figure 1). The incidence of seizures during hospitalization was similar in both patient groups. Brain MR at 7–10 days of life showed serious lesions (Barkovich score 3–4) in 12.2% and 40.0% of patients in Groups A and B, respectively, corresponding to an IRR of 3.28 (95% CI 1.07–10.04; *p* = 0.039).

**Table 1:** Neonatal characteristics.

	**GROUP A**	**GROUP B**	***p***	**Total *N* = 53**
	***N* = 42**	***N* = 11**		
Birth weight, g	3,210 (2,760–3,400)	3,180 (3,050–3,560)	0.44	3,200 (2,820–3,450)
Gestational age, weeks	39.5 (38.0–40.4)	40.0 (38.0–41.0)	0.65	39.7 (38.0–40.7)
Apgar score at 10 min	7 (6–8)	8 (7-8)	0.09	7 (6–8)
Intubation before admission to NICU (*n*, %)	23 (54.8)	2 (18.2)	0.04	25 (47.2)
Lowest pH in 1st h of life	7.01 (6.86–7.11)	7.11 (6.90–7.27)	0.17	7.03 (6.90–7.13)
Lowest BE in 1st h of life	−17.45 (−20.9; −15)	−18.3 (−20.5; −12.8)	0.70	−17.9 (−20.8;−14.7)
Seizures before admission to NICU (*n*, %)	18 (42.9)	4 (40.0)	1	22 (41.5)
RMI at 7–10 days of life:				
Barkovich score (*n*, %) 0	21 (51.2)	3 (30.0)		24 (47.1)
1–2	15 (36.6)	3 (30.0)		18 (35.3)
3–4	5 (12.2)	4 (40.0)	0.16	9 (17.6)
*MBL2* genotype Exon 1 (*n*, %)				
0	3 (7.5)	1(9.1)		4 (7.8)
A0	8 (20.0)	1(9.1)		9 (17.7)
AA	29 (72.5)	9 (81.8)	0.85	38 (74.5)
*MBL2* genotype promoter: (*n*, %)				
XY+XX	17 (47.2)	4 (36.4)		21(44.7)
YY	19 (52.7)	7 (63.6)	0.53	26 (55.3)
MBL at T1 (ng/ml)	1,390 (896–2,462)	1,323 (640–1,855)	0.54	1,378 (896–2,303)
s100 at T1 (pg/ml)	662 (196–7,063)	2,936 (263–6,517)	0.75	795 (196–7,063)

Values are expressed as median (IQR).

Fisher's exact test and Kruskal–Wallis test, as appropriate.

The percentage are calculated on the number of patients with available data.

*TMissing data are not shown*.

MBL and S100B mean levels on admission to the NICU were similar in both groups of neonates. MBL peak levels were higher in carriers of the AA genotype than in carriers of AO or OO genotypes [AA 3580.9 ± 1294.2 ng/ ml vs. AO+OO 1031 ± 1232 ng/ml (*p* < 0.0001)], confirming the association between circulating MBL levels and MBL genotype. There were no significant differences in the distribution of *MBL2* wild-type and variant genotypes between the two groups of neonates.

### *MBL2* Genotypes, MRI Barkovich Score, and Long-Term Outcome

All patients with a pathological MRI (Barkovich score 3–4) and all babies who died were Exon 1 AA (wild-type) genotype carriers (Table 2). Therefore, the wild-type genotype was significantly more frequent among neonates with more severe short-term outcomes. No significant associations between genotype and long-term outcomes were observed; this most likely reflects the early death of some infants with the wild-type genotype, making long-term observation unfeasible.

**Table 2:** Association of brain MRI Barkovich score at 7–10 days or death, neurological outcome with Promoter and Exon1 genotypes.

	Promoter genotypes^*^				**Exon 1 genotypes**					
	**YY**		**XY+XX**			**AA**		**AO+OO**		
	***n***	**%**	***n***	**%**	***p***	***n***	**%**	***n***	**%**	***p***
Barkovich 3–4/death	2	25	6	75		11	100	0	0	
Barkovich 0–1–2	24	61.5	15	38.5	0.115	27	67.5	13	32.5	0.046
Adverse neurological outcome at 6 months	10	38.5	10	52.6	0.379	15	45.5	6	46.2	1.000
Adverse neurological outcome at 12 months	5	22.7	6	37.5	0.471	8	29.6	3	25	1.000

* *In 1 neonate with MRI scores 3–4, the study of the genotype was not done because the blood sample was not available*.

Regarding the relationship between MRI score and long-term outcomes, 9 neonates had a pathological MRI (Barkovich score 3–4) at 7–10 days of life. Seven of these neonates completed the 6-month multidisciplinary follow-up. At the 6-month evaluation, all seven infants (three in Group A and four in Group B) presented with characteristics of adverse neurological outcome. At the 12-month evaluation, the three infants in Group A and three of the four in Group B were classified as having an adverse neurological outcome. The remaining infant in Group B improved and was classified as normal. The risk of adverse neurological outcome at 12 months for children with a pathological MRI (Barkovich score 3–4) at 7–10 days of life was 8.57-fold higher (95% CI 1.73–42.47; *p* = 0.009) than that of children with a Barkovich score of 0. Therefore, a pathological MRI at 7–10 days of life was a strong predictor of long-term neurological outcomes (Supplemental File 3).

### MBL, S100B Plasma Levels, and MRI

Table 3 reports the association between clinical variables, MBL and S100B, and MRI brain damage (Barkovich scores 3 and 4 vs. all lower scores) based on univariate and multivariate analyses. Seizures during hospitalization and no TH treatment were strongly associated with more severe brain damage. An increase in plasma MBL levels was significantly associated with a pathological MRI (Barkovich score 3–4) at 7–10 days of life or death. In particular, for each 100-unit increase in MBL peak levels, the adjusted odds ratio (AOR) of a pathological MRI or death increased 1.10-fold (Table 4).

**Table 3:** Risk of worse MRI at 7–10 days of life (3/4 vs. 1/0–2 of Barkovich score) by ordinal logistic regression analysis.

	**Univariate analysis**				**Multivariate analysis**			
	**OR**	***p***	**[95% CI]**		**AOR**	***p***	**[95% CI]**	
No hypothermia vs. hypothermia	3.39	0.078	0.87	13.15	7.34	0.017	1.43	37.67
Seizures before admission to NICU: yes vs. no	1.88	0.324	0.54	6.62	0.85	0.848	0.16	4.55
Seizures during hospitalization:Yes vs. no	6.70	0.002	2.02	22.23	9.21	0.002	2.22	38.16
BE	0.96	0.518	0.86	1.08	1.00	0.953	0.84	1.17
pH	1.24	0.896	0.05	30.52	3.21	0.603	0.04	258.16
MBL peak level(for increases of 100 units)	1.02	0.299	0.99	1.05	1.02	0.335	0.98	1.06
S100B peak level(for increases of 10 units)	1.00	0.496	0.99	1.00	1.00	0.193	0.99	1.00

*OR, odds ratio; AOD, adjusted odds ratio*.

**Table 4:** Crude and adjusted risk of worse MRI (Barkovich score 3/4) at 7–10 days of life or death.

	**Univariate analysis**				**Multivariate analysis**			
	**OR**	***p***		**[95% CI]**	**AOR**	***p***		**[95% CI]**
No hypothermia vs. hypothermia	5.00	0.032	1.15	21.71	15.43	0.008	2.01	118.24
MBL peak (for increase of 100 units)	1.07	0.01	1.02	1.13	1.10	0.008	1.03	1.18
S100B peak (for increase of 10 units)	1.00	0.387	1.00	1.01	1.00	0.109	0.99	1.00

*OR, odds ratio; AOD, adjusted odds ratio*.

### MBL and S100B Protein by TH

Although some measurements in Group B were missing, this did not appear to change the trend in plasma MBL or S100B levels. MBL levels increased from T1 to T4 in each group of neonates, continuing to rise over 72 h after hypoxia, while protein S100B simultaneously decreased, in both treated and untreated neonates (Table 5).

**Table 5:** MBL and S100B mean values at different time points in the two groups of neonates.

		**Therapeutic Hypothermia**					**Therapeutic Hypothermia**					**(treated vs. not treated)**
		**Yes**					**No**					
		***N***	**Mean**	**[95% CI]**		***p* vs. T1**	***N***	**Mean**	**[95% CI]**		***p* vs. T1**	***p***
**MBL**												
**ng/ml**												
	*T1*	39	1,786	1,337	2,234		8	1,275	368	2,182		0.349
	*T2*	27	2,050	1,558	2,542	0.216	5	1,839	877	2,801	0.053	0.721
	*T3*	25	2,228	1,725	2,731	0.045	3	1,973	929	3,017	0.051	0.670
	*T4*	22	2,488	1,966	3,010	*0.002*	6	3,041	2,103	3,979	*0.001*	0.374
**S100B**												
**pg/ml**												
	*T1*	38	956	553	1,653		8	1,213	367	4,010		0.720
	*T2*	26	127	68	237	*0.001*	5	400	96	1,658	0.129	0.143
	*T3*	25	158	84	299	*0.001*	3	321	56	1,827	0.136	0.470
	*T4*	22	140	72	271	*0.001*	6	105	28	397	*0.001*	0.707

Mean MBL levels at T1–T3 were higher in Group A than in Group B; at T4, the mean value was higher in Group B, but the difference was not statistically significant. S100B levels decreased in both groups from T1 to T2; the levels decreased faster in treated than untreated neonates, with progressive decreases during the first 7–10 days of life (Table 5) (Supplemental Files 4: Figures S1A,B, S2A,B).

## Discussion

The overexpression of inflammatory mediators may be associated with both cerebral damage and poor prognosis following asphyxia (4–6). From our data, compared with other genetic variants, the presence of the *MBL2* wild-type genotype correlated with high plasma MBL levels and was associated with serious brain damage or early death. Complement is activated through various pathways, including the lectin pathway, in which the key activator is MBL. Individuals with the *MBL2* wild-type genotype show high levels and good functioning of circulating MBL, while carriers of the promoter and/or exon-1 variant have low levels and/or abnormal function of this receptor. Individuals with the *MBL2* wild-type genotype also mount a stronger inflammatory response than subjects carrying other variants (34).

Dysregulated levels of MBL in plasma could be a risk factor for the onset of organ failure in pathological contexts such as hypoxia and ischemia–reperfusion conditions. However, the protective or harmful effect of increased or decreased levels of MBL is still controversial, and study results are often contradictory. Effects also seem to vary with patient age. In neonates, carriers of *MBL2* gene variants associated with low MBL levels appear to be at greater risk of infections. However, there is insufficient evidence to indicate that the administration of recombinant MBL is beneficial under these circumstances and may, in fact, be harmful (16–18, 34). Kim et al. evaluated neurodevelopmental outcomes of full-term infants with congenital heart disease undergoing surgery. In those with *MBL2* gene variants and MBL deficiency, neurodevelopmental status, assessed at 4 years by the Child Behavior Checklist, was worse than in infants with normal MBL levels (*p* = 0.025) (35). The authors suggested that this may reflect the negative effect of an increased infection rate in genetically deficient subjects. We reached the same conclusion in a previous study concerning the effect of *MBL2* genotypes on neurological outcomes in preterm infants (36). Surprisingly, in a recent letter, Kim et al. reported that the mutation in exon 1, codon 54 of *MBL2* is protective in babies who underwent single reconstructive ventricle surgery, based on the results obtained for the Psychomotor Developmental Index and Mental Developmental Index at 14 months, which conflicts with their previous findings (37).

In adults who had sustained traumatic brain injury, Longhi et al. observed an early and persistent MBL-positive immunostaining in the injured cortex and, in animal models, MBL immunoreactivity was observed in the injured cortex within 30 min and persisting up to 1 week post-injury. Mice deficient in MBL showed attenuated sensorimotor deficits compared with wild-type mice at 2–4 weeks post-injury. Furthermore, MBL-null mice showed less cortical cell loss at 5 weeks post-injury compared with wild-type mice (38). These observations confirm experimental data by Yager et al. who demonstrated that focal cerebral ischemia/reperfusion in MBL-null mice induced smaller infarctions, better functional outcome, and diminished C3 deposition and neutrophil infiltration than in wild-type mice. Accordingly, reconstitution of MBL-null mice with recombinant human MBL enhanced brain damage. Among 135 patients who had suffered a stroke, those with MBL-sufficient genotypes and high-circulating MBL levels had a 10.85-fold increased risk of adverse outcome at 3 months than individuals carrying MBL-low genotypes (*p* = 0.008) (39, 40).

Genetics may assist in predicting inflammatory-mediated post-asphyxia outcomes. We observed a relationship between *MBL2* genotype and worse short-term, but not long-term, outcomes. This may reflect early death of many infants with the wild-type genotype, resulting in loss to follow-up. Therefore, genetic factors that promote inflammation could have a greater clinical relevance in neonates than adults and deserve further study.

In our patients, risk of brain damage increased as plasma MBL peak levels increased when patients did not receive HT and in the presence of seizures during hospitalization. As expected, MBL levels were higher in individuals with the wild-type genotype than in those with other variants and significantly increased in the first 10 days of life (10). These data also confirm the effect of *MBL2* on phenotype in neonates. In the perinatal period, MBL concentration normally increases during the first week after birth (16–18, 41), regardless of asphyxia. The liver is the main production site of circulating MBL, and there may be an increase of MBL concentration due to inflammation or growth of the liver, or both, as observed for other acute phase proteins (10). MBL has been shown to bind directly to apoptotic cells and necrotic cells and facilitate clearance of these dying cells by phagocytosis. This clearing effect of MBL, by binding to damaged cell sites, could also explain its increased levels at birth. It could also be a defense factor, active against cells, and molecules aberrantly produced in the host and to prevent invasion of the neonate by bacteria present in the extrauterine environment. This increase could, in itself, become harmful, via activation of the complement system in certain situations (42, 43). MBL levels and increases over time in our neonatal patients were similar to those reported in the literature in full-term infants (17).

Mean MBL levels at T1–T3 were slightly higher in neonates who received HT (Group A) compared with those who did not (Group B) even though some values were missing in Group B. At T4, the mean value was higher in the group of untreated neonates and, although not statistically significant, may suggest a faster resolution of inflammation in treated compared with untreated neonates.

Based on our data, hypothermia appeared to have no significant effect on plasma levels of MBL or S100B protein during the acute phase response to asphyxia or on long-term neurological disabilities.

There do not appear to be published data available on trends in MBL plasma levels and hypothermia in humans. Recently, Gong et al. performed a study in an animal model of cardiac arrest, in which animals were resuscitated after 8 min of cardiac arrest, to determine whether mild hypothermia inhibited systemic and cerebral complement activation after resuscitation. They found that C1q, MBL, C3b, C3a, C5a, and other inflammatory mediators were increased after cardiac arrest under normothermia, and mild cooling substantially reduced this increase. These data support the hypothesis that the complement system is activated through classic, alternative, and lectin pathways after ischemia–reperfusion insults, and whole-body mild hypothermia could inhibit systemic and cerebral complement activation (44).

The probability of severe short-term outcomes, such as brain injury and/or death, increased in our study with the progressive increase in MBL peak levels. MBL binds to mannan moieties on microorganisms and surfaces of dying cells, triggering complement system activation via the lectin pathway. This process could exacerbate tissue damage after systemic ischemia/reperfusion, when a specific autoreactive IgM binds to exposed tissue antigens and recruits serum MBL to form an IgM–MBL complex, as demonstrated in experimental models of traumatic brain injuries. The severity of brain damage (i.e., high MRI score) is regulated by the duration and depth of hypoxia, intensity of the inflammatory response, and the amount of tissue involved, but not by the IgM amount (20, 39). The accumulation of pathological substances in response to a traumatic or toxic event in the brain could cause the activation of glial cells, which produce neurotoxic inflammatory cytokines, upsetting the balance between pro-inflammatory and modulating factors and worsening the tissue damage (40).

S100B protein levels decreased substantially 48 h after the hypoxic insult in both groups of neonates, although the reduction was more pronounced in those treated with HT. The serum profile of S100B during the first 72 h following birth has recently been reported in another study in asphyxiated infants treated with TH. S100B levels at baseline and 72 h appeared to strongly predict death or neurological abnormalities at the age of 14 days (28–30). In the patients in our study, S100B levels were high after asphyxia and decreased in the days that followed, more so in treated neonates. These observations may support the measurement of S100B in plasma and/or other biologic fluids to diagnose or monitor the severity of acute brain damage. We did not find any association with long-term adverse neurological outcomes in our patients. Some authors have demonstrated that in newborn infants undergoing TH, the release of S100B could be modulated by treatment, reflecting its therapeutic effect on damaged cells (29). Our study has confirmed this.

In conclusion, in the current study, the *MBL2* wild-type genotype was associated with the highest MBL levels and worst short-term outcomes after hypoxia in TH-treated and untreated neonates. Thus, the wild-type genotype could be associated with poor prognosis. The progressive increase of plasma MBL levels and not treating with TH enhanced the risk of brain damage or death. S100B protein levels decreased, more so in treated than in untreated patients. Effects of asphyxia persisted for hours and days after birth in neonates, and the TH effect on MBL plasma profiles is uncertain. We did not observe any relationship between the *MBL2* genotype and long-term outcomes; however, there was a strong association between a pathological MRI (Barkovich score 3/4) and developmental disabilities. Data from this study are preliminary and warrant further investigation.

## Limitations of the Study

The main limitation of this study was the low number of patients, especially in the group not treated with TH, precluding statistical significance to be reached for some measures. In some cases, blood samples were not available, particularly in neonates not undergoing hypothermia. This was because we only used blood samples from routine clinical practice, choosing not to take additional samples for ethical reasons. In addition, measurements were not carried out on CSF samples, as lumbar puncture is not included in the international protocol for the management of neonates during TH.

## Data Availability Statement

The original contributions presented in the study are included in the article and in the Supplementary files, further inquiries can be directed to the corresponding author/s.

## Ethics Statement

The studies involving human participants were reviewed and approved by Bambino Gesù Children's Hospital Ethics Committee (Protocol No: 1381 OPBG 2017- N°1381, 2017). Written informed consent to participate in this study was provided by the participants' legal guardian.

## Author Contributions

The first draft of the manuscript was written by the first author and no honorarium, grant, or other form of payment was given to anyone to write the manuscript. CA as neonatologist and epidemiologist conceived and designed the study, conceived data analysis, wrote the manuscript and revised the final version. GP and TD as biologists, have performed laboratory exams, significantly contributed to design statistical analysis and to interpret data and she revised the article. RI as biologist, contributed to collect, to store and to catalog biological samples. MM and FN as geneticists, have performed genetical analysis of samples. SB as psychologist, has performed the neurodevelopmental evaluation by Bayley's scales on patients enrolled in the study. VM as neonatologist, significantly contributed to data collection, to draft and to revise the article. DL as expert neuroradiologist, performed the MRI diagnosis and classified exams by Barkovic score. LT and VMD as statisticians have performed data analysis.

## Conflict of Interest

The authors declare that the research was conducted in the absence of any commercial or financial relationships that could be construed as a potential conflict of interest. The handling Editor declared a past co-authorship with several of the authors CA and VMD.

## Abbreviations

MBL: Mannose binding lectin
S100 B: S 100 B protein
TH: Therapeutic hypothermia
NICU: Neonatal intensive care unit
BS/WS: Basal ganglia/watershed.
