# Supplementary material for: Mannose Binding Lectin, S100 B Protein, and Brain Injuries in Neonates With Perinatal Asphyxia
Source: Front Pediatr. 2020 Sep 17;8:527. doi: 10.3389/fped.2020.00527 (PMC7527601; doi:10.3389/fped.2020.00527)
Supplement: Supplementary file 1 [file Table_1.docx]

| Score | Finding |
| --- | --- |
| Basal ganglia/watershed (BG/W) | |
| 0 | Normal |
| 1 | Abnormal signal in basal ganglia or thalamus |
| 2 | Abnormal signal in cortex |
| 3 | Abnormal signal in cortex and basal ganglia (basal ganglia or thalami) |
| 4 | Abnormal signal in entire cortex and basal nuclei |

**Supplemental file 1:** Scoring system for brain involvement in hypoxic ischemic encephalopathy in the newborn.
