# Supplementary material for: Mannose Binding Lectin, S100 B Protein, and Brain Injuries in Neonates With Perinatal Asphyxia
Source: Front Pediatr. 2020 Sep 17;8:527. doi: 10.3389/fped.2020.00527 (PMC7527601; doi:10.3389/fped.2020.00527)
Supplement: Supplementary file 2 [file Table_2.docx]

**Supplemental file 2: Management after discharge from the unit**

**Follow-up**

Children who survived entered a one-year follow up and were monitored for neurological, neurodevelopmental, auditory and visual impairment.

Each child underwent a multidisciplinary assessment, consisting of:

1. Neurological Examination: “severe neurological impairment” defined as a child with severe neuromotor delay or cerebral palsy, diagnosed and classified according to the description of function for each limb in those with abnormal neurologic examination. (Evans et al, 1987)
2. Vision and Hearing Exams: normal vision was defined as the “absence of any detectable pathology of the visual system”, mild abnormal vision as “the presence of a mild impairment that allowed useful vision”, and severe visual impairment as “a child who is functionally blind or perceives light only”. Auditory global function was defined as normal in the “absence of any detectable pathology as explored by auditory brainstem evoked potentials”, as mild if requiring hearing aids, or as severe if functionally deaf (uncorrected even with aids).
3. Evaluation of Child Development**:** Child Development was assessed with the use of Bayley Scales of Infant and Toddler Development 3rd Edition (BSID-III). This standardized test of infant development is age normalized to have a mean of 100 and standard deviation of 15. It consists of three scales; the cognitive and motor scales were used in this study. Development was considered impaired if the scores in either scale were ≤70.
4. Neuroimaging: each neonate underwent brain ultrasound exams and magnetic resonance imaging (MRI) at 6 months of life. At 12 months the MRI was repeated, when necessary.

The presence of a pathological brain impairment on MRI (BG/WS Barkovich score from 1–4) and/or severe neurological impairment and/or anticonvulsant therapy and/or an MDI and/or PDI score ≤70 at 6–12 months was defined as an “adverse neurological outcome”.

**References**

Bayley, N. Bayley scales of infant and toddler development, third edition: Administration manual. San Antonio, TX: Harcourt.2006

Evans P, Alberman E, Johnson A, Mutch L. Standardization of recording and reporting cerebral palsy. Dev Med Child Neurol. 1987; 29: 272-276.
