# Supplementary material for: Mannose Binding Lectin, S100 B Protein, and Brain Injuries in Neonates With Perinatal Asphyxia
Source: Front Pediatr. 2020 Sep 17;8:527. doi: 10.3389/fped.2020.00527 (PMC7527601; doi:10.3389/fped.2020.00527)
Supplement: Supplementary file 3 [file Table_3.docx]

| Supplemental file 3: MRI score at 7–10 days of life and risk of adverse neurological outcome at 12 months follow up* | | | |
| --- | --- | --- | --- |
|  | IRR | P value | 95% CI |
| MRI at 7–10 days of life: 1/2 versus 0 | 2.86 | 0.225 | 0.52–15.60 |
| MRI at 7–10 days of life: 3/4 versus 0 | 8.57 | 0.009 | 1.73–42.47 |
| MRI at 7–10 days of life: 3/4 versus 1/2 | 3.00 | 0.089 | 0.85–10.63 |

*Negative binomial regression
