# Supplementary material for: Mannose Binding Lectin, S100 B Protein, and Brain Injuries in Neonates With Perinatal Asphyxia
Source: Front Pediatr. 2020 Sep 17;8:527. doi: 10.3389/fped.2020.00527 (PMC7527601; doi:10.3389/fped.2020.00527)
Supplement: Supplementary file 4 [file Data_Sheet_1.docx]

**Supplementary Data Sheet: Figures 1a,1b,2a,2b.**

Figure 1a. Mean MBL levels (with 95% CI) at different time points from birth in TH-treated

neonates. Estimates are from a random effect linear regression model.

Figure 1b. Mean MBL levels (with 95% CI) at different time points from birth in TH-untreated neonates. Estimates are from a random effect linear regression model.

Figure 2a. Mean S100 B protein levels (with 95% CI) at different time points from birth in TH-treated neonates. Estimates are from a random effect linear regression model based on the logarithm (base 10) of the raw data.

Figure 2b. Mean S100 B protein levels (with 95% CI) at different time points from birth in TH-untreated neonates. Estimates are from a random effect linear regression model based on the logarithm (base 10) of the raw data.
